# Purinergic P2X7 receptor expression increases in leukocytes from intra-abdominal septic patients

**DOI:** 10.3389/fimmu.2023.1297249

**Published:** 2023-11-29

**Authors:** Helios Martínez-Banaclocha, Carlos García-Palenciano, Laura Martínez-Alarcón, Joaquín Amores-Iniesta, Fátima Martín-Sánchez, Giovanni A. Ercole, Ada González-Lisorge, José Fernández-Pacheco, Piedad Martínez-Gil, Julio Padilla-Rodríguez, Alberto Baroja-Mazo, Pablo Pelegrín, Juan José Martínez-García

**Affiliations:** ^1^ Biomedical Research Institute of Murcia (IMIB), University Clinical Hospital Virgen Arrixaca, Murcia, Spain; ^2^ Unidad de Reanimación, Hospital Clínico Universitario Virgen de la Arrixaca, Murcia, Spain; ^3^ Department of Biochemistry and Molecular Biology B and Immunology, Faculty of Medicine, University of Murcia, Murcia, Spain

**Keywords:** sepsis, inflammation, P2X7 receptor, purinergic signaling, danger signals, leukocytes

## Abstract

Inflammation is a tightly coordinated response of the host immune system to bacterial and viral infections, triggered by the production of inflammatory cytokines. Sepsis is defined as a systemic inflammatory response followed by immunosuppression of the host and organ dysfunction. This imbalance of the immune response increases the risk of mortality of patients with sepsis, making it a major problem for critical care units worldwide. The P2X7 receptor plays a crucial role in activating the immune system by inducing the activation of peripheral blood mononuclear cells. In this study, we analyzed a cohort of abdominal origin septic patients and found that the expression of the P2X7 receptor in the plasma membrane is elevated in the different subsets of lymphocytes. We observed a direct relationship between the percentage of P2X7-expressing lymphocytes and the early inflammatory response in sepsis. Additionally, in patients whose lymphocytes presented a higher percentage of P2X7 surface expression, the total lymphocytes populations proportionally decreased. Furthermore, we found a correlation between elevated soluble P2X7 receptors in plasma and inflammasome-dependent cytokine IL-18. In summary, our work demonstrates that P2X7 expression is highly induced in lymphocytes during sepsis, and this correlates with IL-18, along with other inflammatory mediators such as IL-6, IL-8, and procalcitonin.

## Introduction

Sepsis is the leading cause of mortality in the intensive care units of hospitals worldwide, having an impact on millions of people each year and resulting in the death of between a third and sixth of patients (1). However, effective treatments for sepsis are lacking, mainly due to the variety of abnormal changes in cells, tissues, circulatory, metabolic, and immune systems, which are not well understood (2, 3). The concept of sepsis is linked to the systemic inflammatory response syndrome (SIRS), characterized by a systemic inflammatory response induced by an infection that damages host tissues and causes at least one organ dysfunction (4). However, the late phase of sepsis is characterized by an anti-inflammatory response and immunosuppression. This process was described as a compensatory response to protect the host from the high elevation of cytokines and proinflammatory mediators. Nevertheless, the decrease in the inflammatory response induced by sepsis leads to secondary fatal infections and high mortality (5). Leukocytes from septic patients are prone to undergo changes in their metabolism, which is associated with different immune responses in sepsis. In this regard, leukocytes with a regular inflammatory response show an upregulation of glycolysis. Conversely, immunocompromised leukocytes present defects in both glycolysis and mitochondrial metabolism, while fatty acid oxidation is increased (6, 7). Despite this evidence, there is no consensus on establishing a molecular mechanism in the host that initiates the immunosuppression phase in sepsis. We found that this response could be present very early in the initiation of sepsis (8).

In sepsis, the presence of pathogen-associated molecular patterns (PAMPs) induces a systemic cytokine ‘storm,’ characterized by the elevation of different inflammatory cytokines in the blood, including IL-6, TNF-α, IL-1β, IFN-γ, IL-8, and IL-12 (9). For example, it is known that LPS from Gram-negative bacteria induces endotoxic shock characterized by a systemic hyperinflammatory response (10, 11). Some of the PAMPs are also capable of activating inflammasomes, which are multiprotein complexes formed by a subgroup of intracellular pattern recognition receptors. Inflammasomes lead to the activation of caspase-1, subsequently promoting the release of proinflammatory cytokines such as IL-1β, IL-18, the alarmin high mobility group protein B1 (HMGB1), or mitochondrial DNA, through a specific pro-inflammatory programmed cell death process termed pyroptosis (12–16). The most studied inflammasome is the one formed by the nucleotide-binding oligomerization domain, leucine-rich repeat, and pyrin domain-containing protein 3 (NLRP3), which can be activated in response to different PAMPs or damage-associated molecules, such as extracellular concentrations of the nucleotide adenosine triphosphate (ATP) signaling through the P2X7 receptor (17, 18). We previously found that ATP signaling through P2X7 induces mitochondrial depolarization, which impairs the correct activation of the NLRP3 inflammasome in a specific group of septic patients who accounted for the majority of mortality during sepsis (8). Additionally, we found that P2X7 activation on activated non-classical monocytes from septic patients contributes to CD14 shedding to recognize soluble LPS during infection (19).

However, not only do monocytes play an important role in sepsis, but the strong participation of other immune cell subsets has been reported. CD8^+^ T cells are responsible for inducing organ dysfunction and systemic inflammation during the initial phases of sepsis (2). In addition, the activity of NK cells is highly increased during sepsis, activating the immune response by producing IFN-γ (20). After the early pro-inflammatory phase in sepsis, there is a late phase where T cell function is abolished with the absence of both inflammatory and anti-inflammatory factor production (21, 22). B cells and CD4^+^ T cells decrease during the late phase of sepsis, meanwhile, CD8+ T cell levels do not change during sepsis. This phenomenon could explain T cell exhaustion during sepsis (23, 24). Meanwhile, dendritic cells undergo apoptosis, and T regulatory cells and NK cells could initiate the immunosuppressive phase, along with CD8^+^ T cells and particularly NKT cells, which modulate host responses by producing IL-4, polarizing the remaining CD4^+^ T cells toward a Th2 response (20, 25). It is well documented that some of these peripheral blood mononuclear cells (PBMCs) populations, such as monocytes, T cells, and dendritic cells, can also be activated by ATP through P2X7 signaling (26–28), but the expression of P2X7 during sepsis in these populations have not been investigated.

In this study, we evaluated the amount of P2X7 receptor on the surface of PBMCs from a cohort of intra-abdominal origin septic patients during the early hyperinflammatory phase of sepsis and whether the expression of P2X7 receptor correlates with other clinical diagnostic criteria in sepsis. We found that during sepsis, P2X7 receptor expression is significantly increased in T cells, B cells, NK cells, and T regulatory cells, and it is correlated with the P2X7 expression on the surface of monocytes and with different inflammatory markers, including the plasma concentration of ATP. The percentage of lymphocytes decreased in correlation with inflammatory markers, being inversely proportional to the percentage of cells expressing the P2X7 receptor, which was then found to be soluble in the plasma.

## Materials and methods

### Human samples

Clinical samples were obtained as described in Martinez-Garcia et al., 2019, and the procedures were conducted in accordance with the Declaration of Helsinki. The study and its procedures (reference number PI13/00174) were approved by the clinical ethics committee of the Clinical University Hospital Virgen de la Arrixaca (Murcia, Spain). The samples and data from patients included in this study were provided by the Biobanco en Red de la Región de Murcia, which is part of the Spanish National Biobanks Network (B.000859). Patients diagnosed with intraabdominal sepsis, confirmed by exploratory laparotomy, were included in the study. They exhibited at least two diagnostic criteria for sepsis (fever or hypothermia; heart rate greater than 90 beats per minute; tachypnea, leukocytosis, or leukopenia) and two or more organ dysfunctions. Whole peripheral blood samples were collected after receiving written informed consent from intraabdominal sepsis patients (n = 35, **Table S1**) at the Surgical Critical Unit of the Clinical University Hospital Virgen de la Arrixaca (Murcia, Spain). The samples were collected at 1, 3, 5, and 120 days after the development of sepsis, with the first day defined as the collection within 24 hours of sepsis diagnosis (8).

### PBMCs isolation and stimulation

Human PMBCs were collected using Ficoll Histopaque-1077 (Sigma-Aldrich) and cultured in Opti-MEM reduced serum media (Invitrogen) as described in Martinez-Garcia et al., 2019. For functional assays to study the increase of P2X7, 1x10^5^ PBMCs from healthy donors were left unstimulated or stimulated with IL-2 (20 ng/ml; Preprotech) or LPS O55:B5 (1 µg/ml; Sigma-Aldrich) or PMA (10 ng/ml; Sigma-Aldrich) with or without ionomycin (500 ng/ml; Sigma-Aldrich), antiCD3/CD28 (20 µl/ml; Stemcell Technologies), or pokeweed mitogen (PWM, 1 µg/ml; Sigma-Aldrich) for 48h in 96-wells plates at 37°C and 5% of CO_2_.

### Flow cytometry

P2X7 receptor expression was determined in various subsets of PMBCs from septic patients and analyzed on a FACS Canto analyzer (BD Biosciences), containing the FACS Diva Analysis software (BD Biosciences). Fluorescent-conjugated antibodies for flow cytometry were used in accordance with the gating strategy (**Table S2**; **Supplementary Figures S1, S2, S5**) to determine P2X7 receptor on Treg cells (CD4^+^CD25^+^CD127^-/low^) and on different T cell subset (CD3^+^CD4^+^ and CD3^+^CD8^+^), B cells (CD3^-^CD19^+^), natural killer cells (CD3-CD19^-^CD14^-^CD16^++^), and monocytes (CD3^-^CD14^+^). Monocytes were gated as described in Martinez-Garcia et al., 2019. 5 × 10^5^ cells were blocked for 10 minutes at 4°C using a human Fc-receptor antibody (0.5 μg/ml; BD Biosciences). After blocking, PBMCs were stained for 30 minutes at 4°C with the corresponding conjugated antibody (**Table S2**). For *in vitro* healthy PBMCs stimulation, anti-CD25-PE and antiCD69-FITC were used to determine cell activation. For multiparametric flow cytometry experiments, a fluorescence minus one (FMO) control was included following the recommendations of recent flow cytometry guidelines for immunological studies (29). Cell viability was measured by Propidium Iodide (BD Biosciences) and the FITC Annexin V Apoptosis Detection kit I (BD Biosciences).

### Statistics and data analysis

Statistical analyses were performed using GraphPad Prism 9 (GraphPad Software Inc.). The normality of the samples was determined using the D’Angostino and Pearson omnibus K2 normality test. The non-parametric Mann-Whitney test was used to compare differences between two non-paired groups, and the Kruskal-Wallis test, along with the Dunn’s multiple comparisons test, was used to compare differences between three or more groups. Non-parametric correlations were analyzed using the Spearman correlation coefficient, while parametric correlations were analyzed with the Pearson correlation coefficient. All data are presented as mean values, and error bars represent standard error.

### Editing services

For English editing, the artificial intelligence tool Grammarly was used for grammar checking and correction.

## Results

### The percentages of PBMC populations were altered in septic patients with an elevation of CD4^+^ T cells and a decrease in cytotoxic lymphocytes

**Figure 1:**
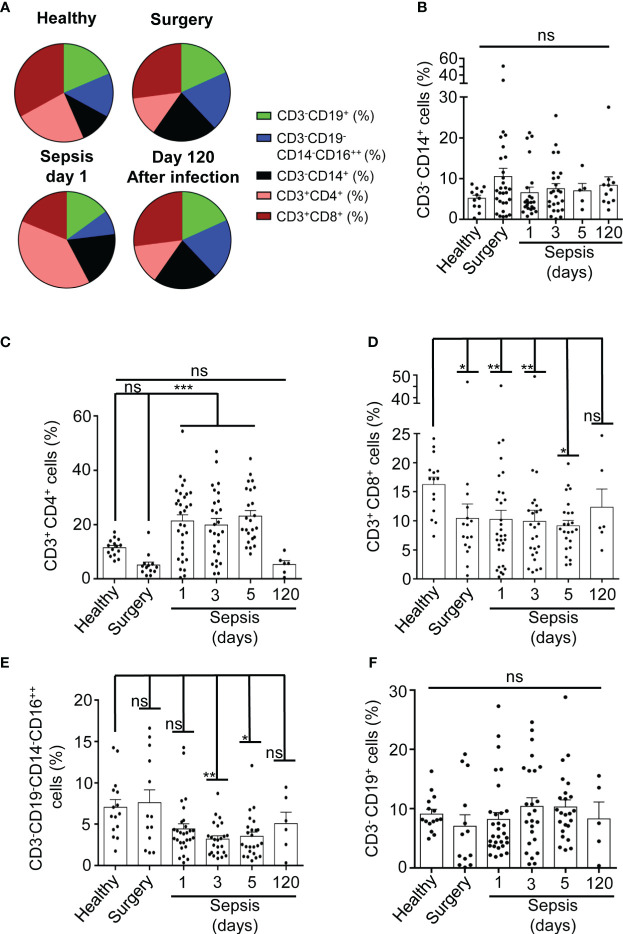
Percentage of PBMCs populations in septic patients. **(A)** Average percentage of populations in sector graphs of gated PBMCs in healthy (n=15) and surgery (n=13) controls, septic patients at day 1 (n=30) or after 120 days of infection (n=6) (as is indicated in the graph). Green, blue, black, magenta and brown were used to indicate the following cell populations: CD3^-^CD19^+^, CD3^-^CD19^-^CD14^-^CD16^++^, CD3^-^ CD14^+^, CD3^+^CD4^+^ and CD3^+^CD8^+^ respectively. **(B-F)** percentage of cells from PBMCs in septic patients at day 1, 3, 5 and 120 as well as in healthy and surgery controls gated for CD3^-^CD14^+^ **(B)**, CD3^+^CD4^+^ **(C)**, CD3^+^CD8^+^ **(D)**, CD3^-^CD19^-^CD14^-^CD16^++^ **(E)**, and CD3^-^CD19^+^ cells **(F)**. Kruskal-Wallis and multiple comparisons Dunn´s test were used for **(B-F)**; P value was measured in each group respect healthy controls; each dot represents a single patient; *p< 0.05; **p< 0.01; ***p< 0.001; ns, not significant difference (p> 0.05).

We initially observed that the percentage of different PBMC populations in our cohort of abdominal surgery septic patients, in comparison with healthy and surgery controls, was affected (**Figure 1A**). The PBMC full population was gated to avoid cell aggregates (**Supplementary Figures S1 A, B, S5A, B**). Percentage of Monocytes, gated as CD3^-^CD14^+^ (8), were separated from lymphocytes by the size (**Supplementary Figures 2A-C**), and did not increase after 24 hours of sepsis development when compared to healthy individuals (**Figures 1A, B**). However, no significant differences were detected when compared with non-septic patients after abdominal surgery (**Figures 1A, B**), suggesting that sepsis did not significantly change the population of monocytes. CD3^+^CD4^+^ T cells significantly increased early in sepsis (P<0,001), and their levels were restored 120 days after infection at similar levels to surgery controls (**Figures 1A, C**). An increase of the proportion of CD3^+^CD4^+^ T cells in sepsis was observed in some septic patients where they could represent up to 50% of total PBMCs. Nevertheless, other patients did not present changes in CD3^+^CD4^+^ T cell proportion concerning controls (**Figure 1C**). In contrast to CD3^+^CD4^+^ T cells, the percentage of CD3^+^CD8^+^ T cells decreased significantly from 24 hours (P< 0,05) and up to 5 days of sepsis development (P<0,01), as was observed after abdominal surgery patients (**Figures 1A, D**). CD3^-^CD19^-^CD14^-^CD16^++^ NK cells presented a similar pattern to the CD3^+^ CD8^+^ cells, presenting a significant decrease at days 3 (P<0,001) and 5 (P<0,05) of sepsis but not after abdominal surgery (**Figures 1A, E**). CD3^-^CD19^+^ B cell proportion was maintained during sepsis (**Figures 1A, F**). Percentage of dead PBMCs was similar in both septic patients and healthy controls (**Supplementary Figures S2D-F**).

### CD3^-^ PBMCs presented a high increase in the percentage of P2X7 receptor expression in sepsis and correlated with proinflammatory markers

**Figure 2:**
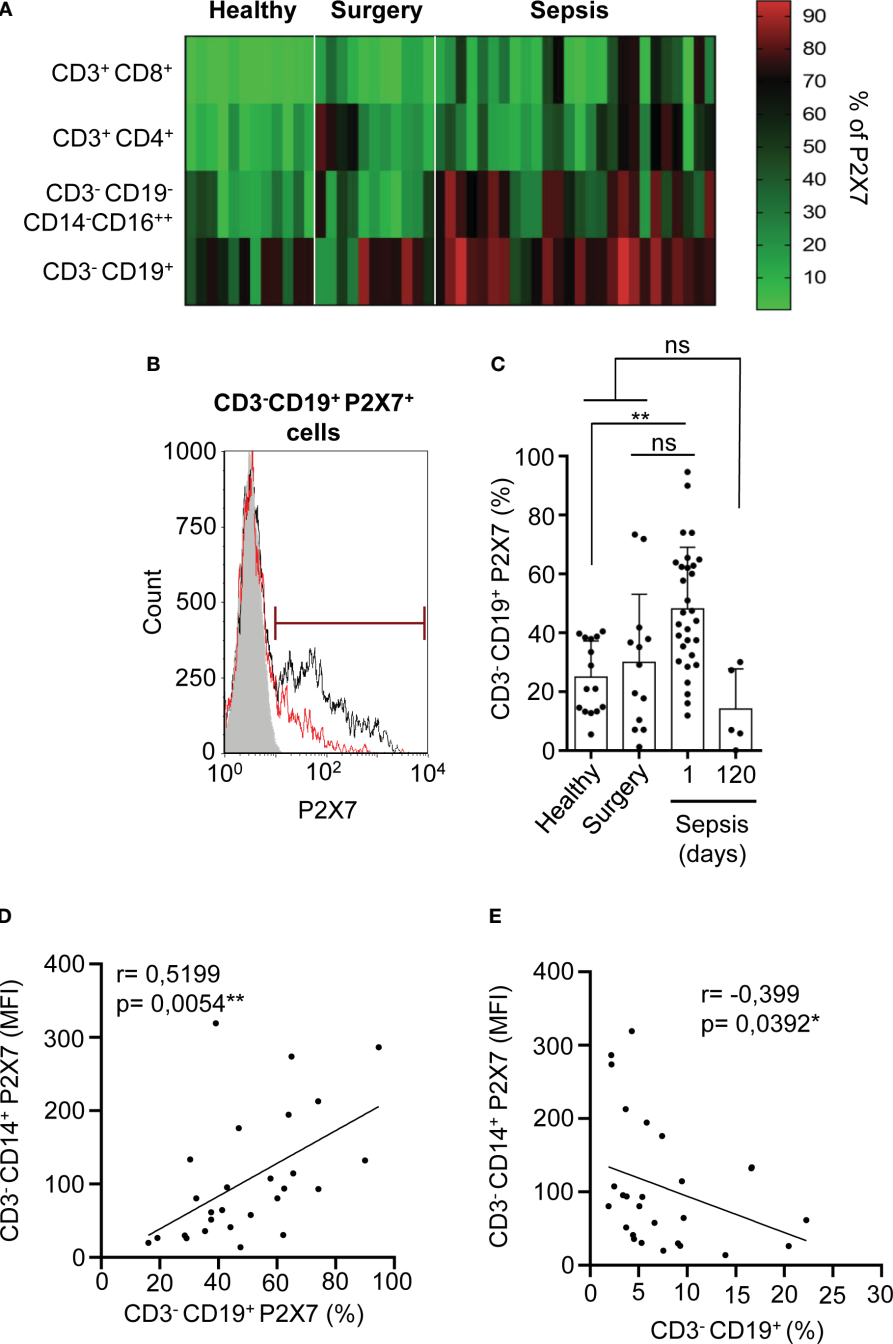
Percentage of P2X7 receptor expression in CD3^–^CD19^+^ cells. **(A)** Heat-map for the percentage of P2X7 receptor-positive cells in different PBMCs populations from septic patients and healthy or surgery controls. Red represents a high percentage of P2X7 receptor cells, meanwhile green represents a low percentage of P2X7 receptor cells. **(B)** Histogram representing the percentage of P2X7 receptor-positive cells in a representative septic patient and healthy control. The grey histogram represents background staining, the red line shows the P2X7 percentage in a healthy control and the black line shows the P2X7 percentage in a septic patient. **(C)** Percentage of CD3^-^CD19^+^ cells stained for P2X7 in septic patients at day 1 and 120, **(D)** Correlation between the P2X7 median intensity fluorescence (MFI) from CD3^-^CD14^+^ cells and the percentage of CD3^-^CD19^+^ cells expressing P2X7, **(E)** or the percentage of CD3^-^CD19^+^ cells. Kruskal-Wallis test and multiple comparisons Dunn´s test were used for **(C)** P value was measured in each group respect healthy controls; for correlations, r Pearson test was used for parametrical distributed variables on **(D)** r Spearman test was used for non-parametrical distributed variables on **(E)** each dot represents a single patient; *p< 0.05; **p< 0.01; ns, not significant difference (p> 0.05).

Our previous work has shown that almost all monocytes are positive for P2X7 receptor expression in both healthy donors and septic patients (8); thus, we then determined the percentage of P2X7 receptor-positive cells in the different PBMCs population from septic patients. We observed that CD3^-^CD19^+^ B cells from septic patients experienced a high increase in P2X7 receptor-positive cells, while P2X7 receptor expression was weakly increased in CD3^+^ T cells (**Figure 2A**). B cells were gated as CD19^+^ cells from the CD3^-^ population (**Supplementary Figures S1C, D**). B cells positive for the P2X7 receptor also presented a higher P2X7 expression in septic patients after 24 hours of infection development when compared to healthy and surgery controls (P<0,01) (**Figures 2B, C**).

The percentage of CD3^-^CD19^+^ B cells expressing the P2X7 receptor was reverted 120 days after sepsis development, returning to normal levels found in healthy controls (**Figure 2C**). We then aimed to establish relationships between the expression of P2X7 receptor in different PBMC populations. We found a positive correlation between the expression of the P2X7 receptor in monocytes and the percentage of CD3^-^CD19^+^ B cells expressing the P2X7 receptor (P=0,0054**) (**Figure 2D**). However, the total percentage of B cells was inversely proportional to the P2X7 expression in CD3^-^CD14^+^ monocytes (P=0,0392*) (**Figure 2E**). We also observed a positive correlation of the total percentage of B cells with the total plasma hemoglobin (P=0,058) (**Supplementary Figure S3A**) and a negative correlation with the acute phase marker procalcitonin (P=0,0149*) (**Supplementary Figure S3B**), suggesting that an increase of the inflammatory response in sepsis affects the presence of B cells.

**Figure 3:**
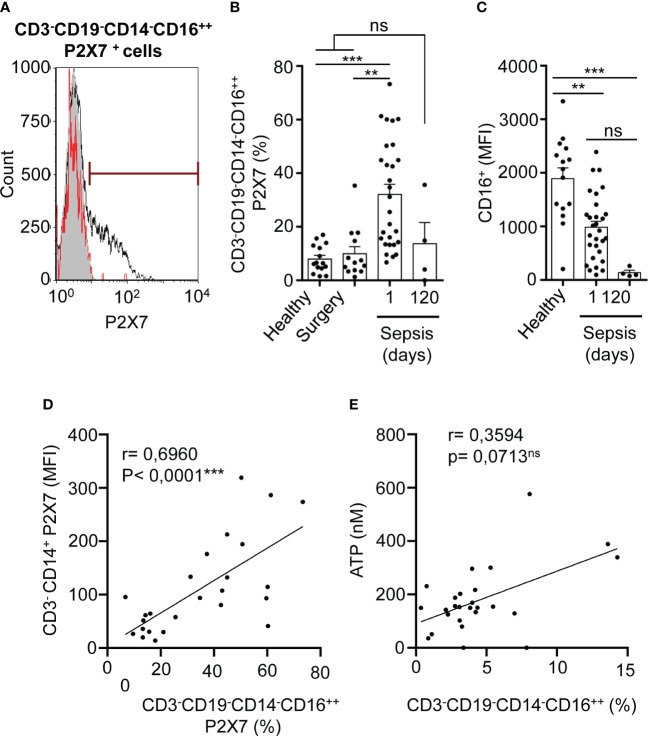
P2X7 receptor expression in NK cells in sepsis. **(A)** Percentage of P2X7 expression in CD3^-^CD19^-^CD14^-^CD16^++^ cells in a representative septic patient and healthy control. The grey histogram represents background staining, the red line shows the percentage of P2X7 expression in a healthy control and the black line shows the percentage of P2X7 expression in a septic patient. **(B)** Percentage of P2X7 from CD3^-^CD19^-^CD14^-^CD16^++^ cells in septic patients at day 1 and 120. **(C)** Expression of CD16 marker on CD3^-^CD19^-^CD14^-^CD16^++^ cells from healthy controls and septic patients at days 1 and 120; **(D)** Correlation between the P2X7 median intensity fluorescence (MFI) from CD3^-^ CD14^+^ cells and the percentage of P2X7 receptor in CD3^-^CD19^-^CD14^-^CD16^++^ cells. **(E)** Correlation between ATP levels and the percentage of CD3^-^CD19^-^CD14^-^CD16^++^ cells; Kruskal-Wallis test and multiple comparisons Dunn´s test were used for **(B, C)** P value was measured in sepsis at day 1 respect healthy donors, surgery controls and 120 days after the infection; for correlations, r Pearson test was used for parametrical distributed variables on D; r Spearman test was used for non-parametrical distributed variables on **(E)** each dot represents a single patient; **p< 0.01; ***p< 0.001; *ns*, not significant difference (p> 0.05).

NK cells (CD3^-^CD19^-^CD14^-^CD16^++^) were gated as CD19^-^ cells from CD3^-^ cell population that were highly positive for CD16 marker in a CD14-CD16 dot-plot, where CD14^-^CD16^++^ cells were selected as NK cells (**Supplementary Figures S1C, E**) (30). P2X7 receptor-positive NK cells were also highly increased in septic patients in comparison with both healthy controls (P<0,001***) and abdominal surgery (P<0,01**) patients and returned to basal levels 120 days after sepsis (**Figures 3A, B**). The CD16 marker in CD3^-^CD19^-^CD14^-^CD16^++^ cells decreased in septic patients after 120 days of recovery (P<0,001***), being smaller than in healthy controls (**Figure 3C**). The percentage of NK cells expressing P2X7 receptor also correlated positively with the expression of P2X7 receptor in CD3^-^CD14^+^ monocytes (P<0,0001***) (**Figure 3D**). We observed a high dispersion on ATP concentration in plasma of septic patients in comparison with healthy controls (**Supplementary Figure S4**), thus we found that the percentage of total NK cells possess a tendency to correlate positively together with the concentration of ATP in plasma (P=0,0713) (**Figure 3E**), but negatively correlated with the acute phase protein procalcitonin (P<0,0001***) (**Supplementary Figure S3C**).

### CD3^+^ PBMCs presented a small increase of the percentage of P2X7 receptor expression in sepsis

**Figure 4:**
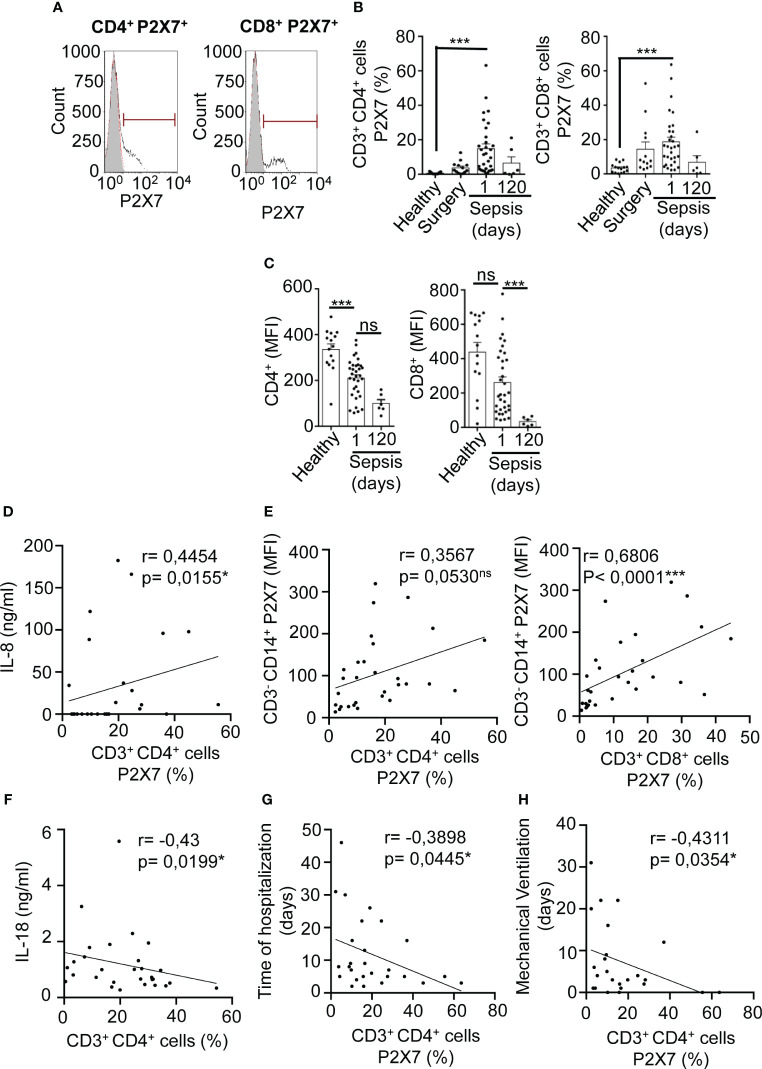
Expression of P2X7 receptor in T lymphocytes from septic patients. **(A)** P2X7 gating of CD3^+^CD4^+^ (left panel) and CD3^+^CD8^+^ (right panel) as indicated. The grey histogram represents background staining, the red line shows P2X7 expression in a healthy control and the black line shows P2X7 expression in a septic patient. **(B)** Percentage of CD3^+^CD4^+^ cells positive for P2X7 (left panel) or CD3^+^CD8^+^ cells positive for P2X7 (right panel) from septic patients at days 1 and 120. **(C)** CD4 marker expression on CD3^+^CD4^+^ cells (left panel) and CD8 expression on CD3^+^CD8^+^ cells (right panel) from healthy controls and septic patients at day 1 or 120 after infection **(D)** Correlation between the IL-8 levels in plasma from septic patients at day 1 and percentage of P2X7 expression in CD3^+^CD4^+^ cells; **(E)** Correlation between the P2X7 median intensity fluorescence (MFI) from CD3^-^CD14^+^ cells and the percentage of P2X7 receptor in CD3^-^CD4^+^ cells (left panel), or percentage of P2X7 receptor in CD3^-^CD8^+^ cells (right panel); **(F)** Correlation between IL-18 levels in plasma from septic patients at day 1 and the percentage of CD3^+^CD4^+^ cells; **(G, H)** Correlations between the percentage of P2X7 receptor expression in CD3^-^CD14^+^ cells and the days of hospitalization in the reanimation unit **(G)** or days of mechanical ventilation **(H)**; Kruskal-Wallis test and multiple comparisons Dunn´s test were used for **(B, C)** P value was measured in sepsis at day 1 respect healthy donors, surgery controls and 120 days after the infection; for correlations, r Pearson test was used for parametrical distributed variables on E; r Spearman test was used for non-parametrical distributed variables on **(D–G)** each dot represents a single patient; *p< 0.05; ***p< 0.001; ns, not significant difference (p> 0.05).

CD3^+^ T cells were plotted in a CD4-CD8 dot-plot to obtain CD3^+^CD4^+^ and CD3^+^CD8^+^ cells (**Supplementary Figures S5C-E**). We found that the percentage of T cells with P2X7 receptor expression increased in both T helper (CD3^+^CD4^+^) and T cytotoxic (CD3^+^CD8^+^) populations in septic patients when compared to healthy controls (P<0,001***), but not to surgery controls (**Figures 4A, B**). The percentage of P2X7 expressing T cells returned to normal values after 120 days of infection when the patients were completely recovered (**Figure 4B**). The level of CD4 and CD8 expression, calculated as the mean of fluorescent intensity, decreased after 120 days of infection in CD4^+^ T and CD8^+^ T cells, respectively, in comparison with 24 hours after sepsis development (P<0,001***) while CD4 and CD8 expression was higher in healthy controls (**Figure 4C**). This suggests that after sepsis recovery, CD3^+^ T cells present a decrease of CD4 and CD8, which could be involved in an impairment of CD3^+^ T cell activation after sepsis. In support of this hypothesis, we observed a positive correlation between the systemic concentration of IL-8 and the percentage of CD4^+^ T expressing the P2X7 receptor (P=0,0155*) (**Figure 4D**). Also, the proportion of P2X7 positive CD4^+^ T and CD8^+^ T cells correlated with the expression of P2X7 receptor in monocytes from septic patients (P=0,053 and P<0,0001***) (**Figure 4E**). However, the percentage of total CD4^+^ T cells was inversely proportional to plasma concentrations of IL-18 (P=0,0199*) (**Figure 4F**), IL-6 (P=0,0418*), IL-8 (P=0,0012**) (**Supplementary Figure S6A**), bilirubin (P=0,0111*), and lactate (P=0,0007***) (**Supplementary Figure S6B**). Similarly, CD8^+^ T cells negatively correlated with plasma concentrations of IL-6 (P=0,0019**) and IL-8 (P=0,0227*) (**Supplementary Figure S7A**), as well as the age of the patient (P=0,0307*) (**Supplementary Figure S7B**). Additionally, we observed that patients that were hospitalized for a long time in the reanimation unit and those patients requiring more days of mechanical ventilation presented a lower percentage of CD4^+^ T cells expressing P2X7 (P<0,05*) (**Figures 4G, H**).

**Figure 5:**
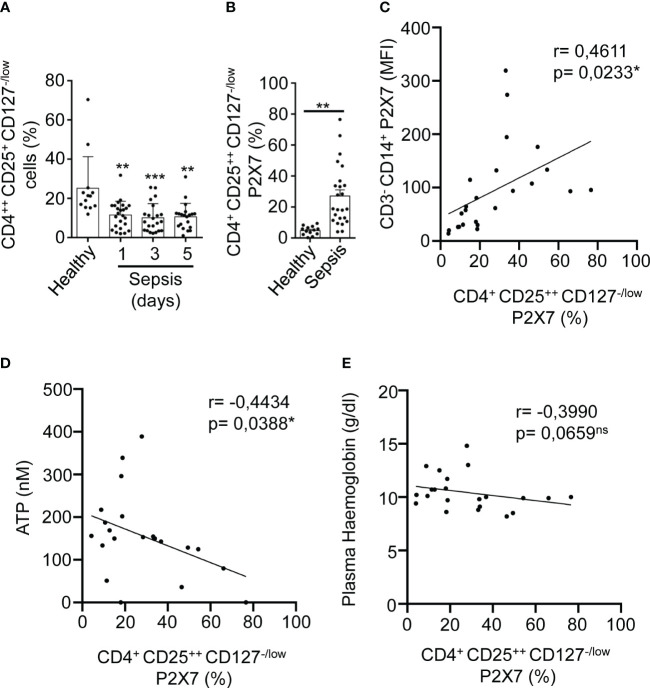
Expression of P2X7 receptor in T regulatory cells from sepsis. **(A)** Percentage of CD4^++^CD25^+^CD127^-/low^ cells from PBMCs in septic patients at days 1, 3, and 5 as well as in healthy controls. **(B)** Percentage CD4^++^CD25^+^CD127^-/low^ cells positive for P2X7 from septic patients at day 1. **(C)** Correlation between the P2X7 median intensity fluorescence (MFI) from CD3^-^CD14^+^ cells and the percentage of P2X7 receptor in CD4^++^CD25^+^CD127^-/low^ cells, **(D)** Correlations between the percentage of P2X7 receptor expression in CD4^++^CD25^+^CD127^-/low^ and ATP levels, **(E)** or haemoglobin levels in plasma from septic patients at day 1; Kruskal-Wallis test and multiple comparisons Dunn´s test were used for **(A)** Mann-Whitney test was used for **(B)** P value was measured in each group respect healthy controls; for correlations, r Pearson test was used for parametrical distributed variables on **(C)** r Spearman test was used for non-parametrical distributed variables on **(D, E)** each dot represents a single patient; *p< 0.05; **p< 0.01; ***p< 0.001; ns, not significant difference (p> 0.05).

T regulatory (Treg) cells were gated from PBMCs cells in a CD4-CD25 dot-plot, where CD4^++^CD25^+^ cells were selected to analyze the expression of P2X7 (**Supplementary Figures S5A, B, F**). Once gated in CD127^-/low^ P2X7 receptor-expressing cells in sepsis, we observed that P2X7 receptor expression increased even in more than 50% of CD4^++^CD25^+^CD127^-/low^ cells (**Supplementary Figure S5G**). However, the number of CD4^++^CD25^+^CD127^-/low^ Treg decreased during sepsis (P<0,01**) (**Figure 5A**). Similar to other cell subsets, the percentage of CD4^++^CD25^+^CD127^-/low^ cells with P2X7 expression was increased in sepsis (P<0,01**) (**Figure 5B**), and positively correlated with the expression of the P2X7 receptor in monocytes (P=0,0233*) (**Figure 5C**). Nevertheless, the percentage of CD4^++^CD25^+^CD127^-/low^ P2X7^+^ cells decreased when compared to the plasma ATP (P=0,0388*) (**Figure 5D**) and haemoglobin concentrations (P=0,0659) (**Figure 5E**). The percentage of expression of the P2X7 receptor in CD3^+^CD4^+^ and CD3^+^CD8^+^ T cells in septic patients correlated with the percentage of P2X7 receptor expression in CD4^++^CD25^+^CD127^-/low^ Treg cells (P<0,05*) (**Supplementary Figures S8A, B**), but this correlation was not found in healthy controls. Additionally, P2X7 receptor expression in Treg cells was inversely proportional to bicarbonate concentration (P=0,0565) (**Supplementary Figure S9**).

Although we showed that some acute phase and proinflammatory markers positively correlated with the percentage of lymphocyte subsets expressing P2X7, we were not capable of finding a significant correlation between the lymphocyte subsets’ proportions and the expression of P2X7 receptor (**Supplementary Figures S10A-E**). However, almost all these correlations presented a negative slope, and for NK cells and T regulatory cells, the negative correlation was close to significant (P=0,0884 for NK cells and P=0,073 for T regulatory cells) (**Supplementary Figures S10B, E**). This could relate high P2X7 receptor expression with the decrease in cell number, in line with the cytolytic activity of this receptor (31, 32).

### P2X receptor expression is involved in the communication of monocytes, T cells and B cells

**Figure 6:**
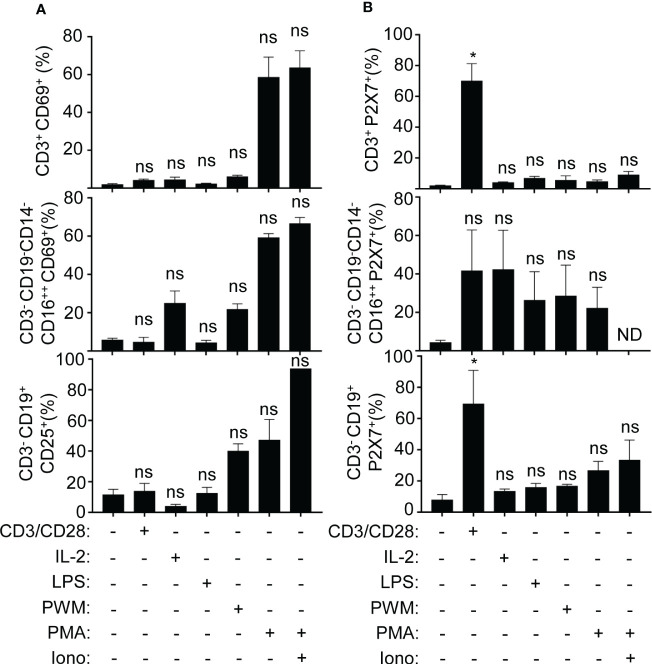
Classical CD3 activation induces the P2X7 expression in T and B cells. **(A)** Percentage of the expression of activation markers CD69 (for T cells and NK cells) and CD25 (for B cells), **(B)** Percentage of P2X7 expression on CD3^+^, CD3^-^CD16^++^, CD3^-^CD19^+^, all of them stimulated with CD3/CD28 antibody, recombinant IL-2, LPS, PWM, and PMA with or without ionomycin or without simulation. Kruskal-Wallis test and multiple comparisons Dunn´s test were used for **(A, B)** P value was measured in each group respect healthy controls; *p< 0.05; ns, not significant difference (p> 0.05).

As P2X7 receptor expression increases in different PBMC populations during sepsis, we then aimed to evaluate the expression of the P2X7 receptor in PBMCs from healthy volunteers in unstimulated and treated cells with different T cell, B cell, and NK cell activators (**Figure 6B**). In parallel, we evaluated the activity of those PBMCs with the CD25 and CD69 activation markers for B and T cells, respectively, for the same *in vitro* treatments (**Figure 6A**) (33–36). Only anti-CD3/CD28 stimulation was able to significantly induce the expression of the P2X7 receptor in CD3^+^ T cells and in CD3^-^CD19^+^ B cells (P<0,05), but not in CD3^-^CD19^-^CD14^-^CD16^++^ NK cells (**Figure 6B**). The percentage of total cell populations did not change upon stimulation (**Figure 6A**).

As the percentage of P2X7 receptor-positive cells increase in the main PBMCs populations during sepsis, we further found that, contrarily to healthy donors, the percentages of P2X7 receptor-positive cells strongly correlated between the different PBMCs populations in septic patients (**Supplementary Figures S11A, B**). The increase of P2X7 receptor expression in CD3^-^CD14^+^ monocytes and the percentage of P2X7 expressing CD3^+^CD8^+^ cells correlated with the increase of P2X7 receptor-positive cells in the rest of PBMCs populations (**Supplementary Figure S11A**). The increase of P2X7 receptor-positive cells in CD3^-^CD19^+^ and CD3^-^CD19^-^CD14^-^CD16^++^ cells positively correlated between them in addition to CD3^-^CD14^+^ and CD3^+^CD8^+^ cells (**Supplementary Figure S11A**). This observation could suggest the importance of the communication between monocytes and lymphocytes in the coordinated inflammatory response against infections by increasing the P2X7 receptor in these different populations (37). On the contrary, in healthy controls, a weak correlation between the percentage of P2X7 receptor expression in CD3^+^CD4^+^ and CD3^+^CD8^+^ T cells was observed (**Supplementary Figure S11B**).

**Figure 7:**
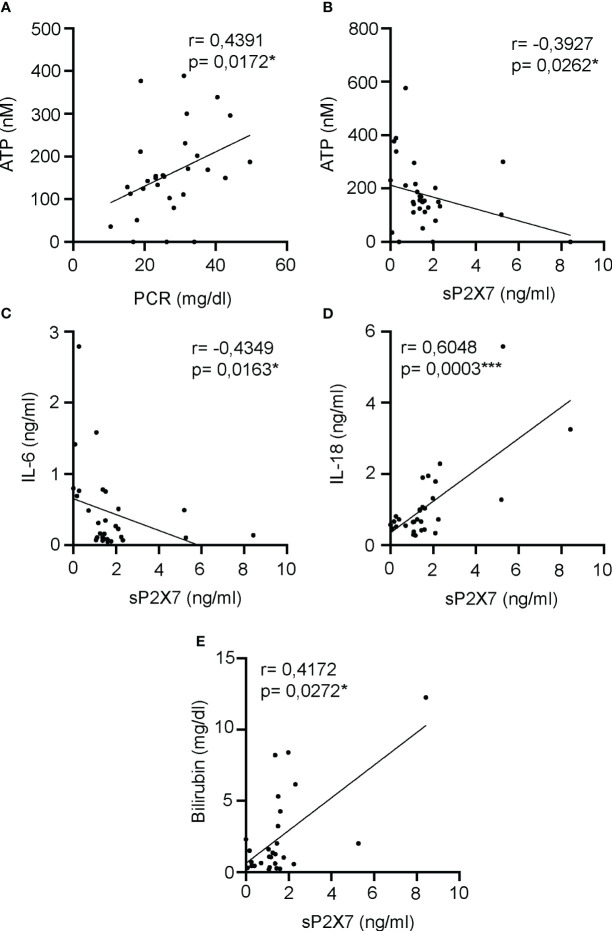
Correlations between soluble P2X7 receptor and inflammatory markers in plasma of septic patients. **(A, B)** Correlation between the plasma levels of ATP and acute phase inflammatory marker PCR **(A)** or soluble P2X7 receptor **(B)** in septic patients at day 1. **(C-E)** Correlations between the soluble P2X7 receptor in plasma of septic patients and the concentrations of IL-6 **(C)**, IL-18 **(D)** or bilirubin **(E)**. r Spearman test was used for non-parametrical distributed variables; each dot represents a single patient; *p< 0.05; ***p< 0.001.

We have observed a negative correlation between the percentage of CD3^-^CD19^-^ CD4^++^CD25^+^CD127^-/low^ cells which express P2X7 and receptor, and plasma ATP, meanwhile we found a positive correlation with the total proportion of CD3^-^CD19^-^CD14^-^CD16^++^ cells. We wondered if plasma ATP kept any relationship with the soluble P2X7 receptor and other soluble clinical markers for the acute phase of sepsis in the plasma of septic patients. We found that plasma ATP concentration positively correlated with the C-reactive protein (CRP) (P=0,0172*) (**Figure 7A**), but negatively correlated with the soluble P2X7 receptor in plasma (P=0,0262*) (**Figure 7B**). The soluble P2X7 receptor also negatively correlated with IL-6 concentration (P=0,0163*) (**Figure 7C**), and positively correlated with the inflammasome-dependent cytokine IL-18 (P=0,0003***) (**Figure 7D**) and bilirubin (P=0,0272*) (**Figure 7E**).

In summary, we found that the percentage of P2X7 expressing PBMCs is increased during the early inflammatory response of sepsis, while the proportion of these cell populations decrease. This effect positively correlated with the P2X7 expression in CD3^-^CD14^+^ monocytes for all PBMCs populations, and in some cases together with other inflammatory and tissue damage markers. Furthermore, our *in vitro* studies revealed that T cell activation induces the increase of P2X7 receptor expression in both CD3^+^ and CD3^-^CD19^+^ cells. Our data suggests a communication between monocytes, T and B cells. Additionally, we found that elevated soluble P2X7 receptor in plasma correlated with the inflammasome-dependent release of IL-18 in sepsis.

## Discussion

In our study, we found an elevated percentage of lymphocytes that expressed the P2X7 receptor on the cell surface in patients with sepsis when compared to healthy controls. The percentage of P2X7 receptor-expressing T, B, and NK cells returned to normal values after recovery from sepsis. This data suggests that T and B cells increase P2X7 receptor expression during sepsis, as we also demonstrated *in vitro* following CD3/CD28 activation. This increase in P2X7 receptor-expressing lymphocytes was paralleled by an increase in soluble P2X7 receptor in the plasma from septic patients (8).

Previous studies have shown that B and CD4^+^ T cells undergo apoptosis during sepsis, leading to an increase in the proportion of cytotoxic NK and CD8^+^ T cells (23). In contrast to this observation, the percentage of CD4^+^ T cells increased in the blood of our cohort of septic patients, while the percentage of B cells did not change, and the percentage of both cytotoxic CD8^+^ T cells and NK cells in our cohort of septic patients decreased. Unlike our study, these reports did not differentiate patients by sepsis origin, making it difficult to assess the initial day of sepsis. Furthermore, these reports examined septic samples during the late phase of the disease (ranging from 6 up to 70 days) (23); whereas our study primarily focuses on the early phase, during the first 24 hours (and up to 5 days) after sepsis initiation. Other studies have shown that CD4^+^ T cells decrease only in septic survivors after 6 days of sepsis development compared to control groups (38). This finding is further supported by other studies that indicate high levels of cell death in the late state of sepsis, reducing the number of circulating lymphocytes (39). However, our data suggests that the decrease in lymphocyte proportion initiates in the early phase of sepsis with the onset of the inflammatory response, which may continue accumulating until the late phase of sepsis, where the cytolytic activity of the P2X7 receptor may contribute to lymphocyte death. In this regard, the inflammatory response in the early phase of sepsis may play a role in the recovery in the late phase of sepsis. However, the decrease in lymphocytes could be a double-edged sword, as an exacerbated cytolytic activity of P2X7 during the early phase of sepsis could contribute to immunoparalysis in the late phase of sepsis. Thus, alternative factors may participate in regulating cell death induced by the lytic effect of the P2X7 receptor. For example, previous studies have shown that, towards the end of the early hyperinflammatory phase of sepsis, the P2X7 receptor competes with several ectoenzymes, such as CD39, which is produced in monocytes and scavenges extracellular ATP, contributing to the reduction of P2X7 receptor activity (40, 41).

The death of activated T, B, and NK cells during the acute inflammatory phase of sepsis decreases the patient’s response to secondary infections in the late phase. Therefore, patients with a higher percentage of lymphocytes expressing the P2X7 receptor correlate with P2X7 expression in monocytes, as well as different inflammatory mediators such as IL-8, CRP, or procalcitonin. However, they also correlate with the decrease in total lymphocytes, suggesting that P2X7-expressing cells are more susceptible to death, resulting in a higher soluble P2X7 receptor in the plasma.

Additionally, we observed that P2X7 was expressed in a high percentage of lymphocytes from septic patients, including Treg cells. This observation is related to a previous report that demonstrated the high sensitivity of Treg cells to extracellular ATP mediated by P2X7 receptor signaling (42). Our *in vitro* studies showed that CD4^+^ T cell activation could induce the expression of P2X7 in B cells. Furthermore, a negative correlation was found between the percentage of P2X7 expressing CD4^+^ T cells and the increasing number of days hospitalized in the reanimation unit and the increasing number of days with mechanical respiratory assistance. We observed that the total percentage of CD4+ T cells decreased in septic patients with higher values of the proinflammatory cytokine IL-18 in plasma. Accordingly, we also observed a decrease in the expression of CD4 during sepsis, which is essential for T cell recognition by the antigen-presenting cell (43, 44). Taken together, these results demonstrate the importance of CD4^+^ T cell function during sepsis (22), which positively correlates with the increase of P2X7 receptor in these cells.

We did not find any correlation when we compared P2X7 expression from both cytotoxic CD8^+^ T cells and NK cells with the percentage of P2X7 expressing CD4^+^ T cells, and the increase of CD4^+^ T cells did not negatively correlate with the decrease of both cytotoxic populations. This may suggest that the role of the P2X7 receptor on CD4^+^ T cells is likely independent of the increase of the P2X7 receptor in cytotoxic lymphocytes. Notably, we observed a decrease of both CD8 and CD16 markers for CD8^+^ T and NK cells, respectively, in sepsis, which could be associated with the high expression of the P2X7 receptor, as was previously observed for the monocytic marker CD14 in sepsis (19).

We demonstrated that higher levels of soluble P2X7 in plasma from septic patients are negatively related to plasma ATP, IL6 and IL8. We can hypothesize that after the early phase of sepsis, where inflammatory markers such as IL-8, IL-6, CRP, or procalcitonin are highly elevated, ATP is also released as a danger signal. As a potential lytic effect, P2X7 could be released after cell death (31, 32). However, the role of the extracellular P2X7 receptor is not fully understood, and it could potentially be exclusively a released cellular particle due to exacerbated cell death.

In conclusion, we report an increase in the P2X7 receptor in different lymphocytes from septic patients, positively correlating with increased inflammatory mediators. This increase in P2X7 receptor is concomitant with an immunosuppression-like phase characterized by decreased numbers of lymphocytes and higher levels of soluble P2X7 in plasma. The higher percentage of P2X7 receptor in septic lymphocytes and its potential relationship with lymphocyte activity in sepsis open the possibility of P2X7-blocking therapies as a potential treatment for sepsis to improve lymphocyte survival.

## Data availability statement

The raw data supporting the conclusions of this article will be made available by the authors, without undue reservation.

## Ethics statement

The studies involving humans were approved by clinical ethics committee of the Clinical University Hospital Virgen de la Arrixaca. The studies were conducted in accordance with the local legislation and institutional requirements. The participants provided their written informed consent to participate in this study.

## Author contributions

HM-B: Investigation, Formal Analysis, Writing – review & editing. CG-P: Methodology, Investigation, Writing – review & editing. LM-A: Investigation, Writing – review & editing. JA-I: Investigation, Writing – review & editing. FS: Investigation, Writing – review & editing. GE: Investigation, Writing – review & editing. AG-L: Investigation, Writing – review & editing. JG-P: Investigation, Writing – review & editing. PM-G: Investigation, Writing – review & editing. JP-R: Investigation, Writing – review & editing. AB-M: Investigation, Writing – review & editing. PP: Funding acquisition, Methodology, Supervision, Writing – review & editing. JM-G: Funding acquisition, Methodology, Conceptualization, Investigation, Formal Analysis, Visualization, Writing – original draft, Writing – review & editing.
