# Supplementary material for: Purinergic P2X7 receptor expression increases in leukocytes from intra-abdominal septic patients
Source: Front Immunol. 2023 Nov 29;14:1297249. doi: 10.3389/fimmu.2023.1297249 (PMC10716420; doi:10.3389/fimmu.2023.1297249)
Supplement: Supplementary file 1 [file DataSheet_1.pdf]

Supplementary information for:

Purinergic P2X7 receptor expression increases in leukocytes from sepsis

Martínez-Banaclocha, et al.

Contents:

- Supplementary Methods
- Supplementary Tables
- Supplementary Figure legends
- Supplementary Figures

Supplementary Methods

Clinical data

Clinical markers for sepsis were determined within the first 24 h of sepsis development at the surgical critical unit: CRP, PCT, and the NT-ProBNP were detected as molecular markers of acute phase of sepsis. Other cellular and biochemical markers were analysed in blood samples, such as the plasma concentration of lactate, bicarbonate, creatinine, bilirubin, and haemoglobin.

ATP and cytokines determination

Plasma from septic patients and control groups (healthy individual and abdominal surgery patients) was used to measure the concentration of human IL-1b, IL-8, and IL-6 using the CBA Flex Set kit (BD Biosciences), following the manufacturer instructions. Samples were analysed by flow cytometry using the FCAP Array Software (BD Biosciences). ELISA for Human IL-18 (MBL) and Human soluble P2X7 receptor (Cusabio), as well as ATP determination by FLAAM assay mix (Sigma Aldrich) were measured in plasma of septic patients and control groups by independent assays following manufacturer protocols.

P2X7R antibody conjugation with APC fluorochrome

The Mouse monoclonal anti-human P2X7R (Glaxo SmithKline) was conjugated with the APC fluorochrome using the LYNX Rapid APC conjugation kit (Bio-Rad Laboratories) by following the manufacturer instructions.

Supplementary Tables:

	Healthy controls	Abdominal surgery	Intra-abdominal origin septic patients
**N**	11	14	35
**Age**, mean (range) ± SD	67.45 (49-79) ±10.68	66,29 (38-94) ± 16,03	69.33 (43-91) ±13.34
p value *vs* septic group	*p*> 0.05*ns*	*p*> 0.05*ns*	
**Gender**, N (%)			
Male	5 (45%)	8 (57%)	21 (60%)
Female	6 (55%)	6 (43%)	14 (40%)
p value *vs* septic group	*p*> 0.05*ns*	*p*> 0.05*ns*	

Table S1. Demographic and clinical characteristics of the individuals included in this study

Name	Clone	Dilution	Company
Mouse monoclonal anti-human CD127-FITC	HIL-7R M21	1:200	BD biosciences
Mouse monoclonal anti-human CD16-PE-Cy7	3G8	1:500	BD biosciences
Mouse monoclonal anti-human CD19-PerCP-Cy5.5	HIB19	1:500	Tonbo Biosciences
Mouse monoclonal anti-human CD4-PerCP-Cy5.5	OKT4	1:500	Tonbo Biosciences
Mouse monoclonal anti-human CD25-PE	BC96	1:500	Tonbo Biosciences
Mouse monoclonal anti-human CD69-FITC	FN50	1:500	BD Biosciences
Mouse monoclonal anti-human CD3-FITC	Hit3a	1:500	Tonbo Biosciences
Mouse monoclonal anti-human CD8-PE	OKT8	1:500	Tonbo Biosciences
Mouse monoclonal anti-human CD14-APC-H7	G46-6	1:500	Tonbo Biosciences
Mouse monoclonal anti-human P2X7R	L4	1:5000	Glaxo SmithKline

Table S2. Relation of used antibodies for flow cytometry determination.

Supplementary figure legends.

Figure S1. Gating of CD3$^-$ cells subsets from PBMCs of septic patients. (A) Representative dot-plot for singlets gating in human septic samples. (B) PBMCs gating. (C) CD3$^-$ cells gating (green gate) from PBMCs plot in a septic dyed sample (left panel) and CD3 isotype control (right panel). (D) CD3$^-$ CD19$^+$ cells gating (red gate) from CD3$^-$ plot in a septic dyed sample (left panel) and CD19 isotype control (right panel). (E) CD14$^-$ CD16^{++} cells gating from CD19$^-$ plot in a septic dyed sample (left panel) and CD16 isotype control (right panel).

Figure S2. Gating of Monocytes from PBMCs of septic patients and cell viability. (A) Representative dot-plot for PBMCs gating. (B) Monocytes gating from PBMCs plot. (C) CD14 gating from high SSC CD3$^-$ plot in a septic dyed sample (upper panel) and CD14 isotype control (bottom panel). (D, E) Percentage Annexin V positive cells in representative samples (D) of healthy controls (left panel) and septic patients (right panel) or in both groups (E). (F) Percentage of Propidium Iodide in a healthy control (left panel) and a septic patient (right panel); Mann-Whitney test was used for E; each dot represents a single patient; *ns*, not significant difference.

Figure S3. Correlations between the percentage of CD3$^-$ lymphocytes and clinical data from septic patients. (A) Correlation between the percentage of CD3$^-$ CD19$^+$ cells and plasma haemoglobin, (B) or procalcitonin levels in plasma from septic patients at day 1. (C) Correlation between the percentage of CD3-CD19-CD14-CD16++ cells procalcitonin levels in plasma from septic patients at day 1. r spearman test was used for non-parametrical correlations; each dot represents a single patient; *p< 0.05; ***p< 0.001; *ns*, not significant difference (p> 0.05).

Figure S4. ATP concentration in plasma. Concentration of ATP in plasma of septic patients at day 1 and day 120 after sepsis development, and on healthy and abdominal

surgery controls; Kruskal-Wallis test was used; each dot represents a single patient; *ns*, not significant difference.

Figure S5. Gating of CD3^{+} cells subsets from PBMCs of septic patients. (A) Representative dot-plot for singlets gating in human septic samples. (B) PBMCs gating. (C) Gating for CD3^{+} cells (red and orange gate) from PBMCs plot. (D, E) CD3^{+}CD4^{+} (Orange gate) and CD3^{+}CD8^{+} gating (red gate) from CD3^{+} plot in a septic dyed sample (D) and in CD4 and CD8 isotypes controls (E). (F) CD4^{++} CD25^{+} cells gating (blue gate) from CD3^{+} plot in a septic dyed sample (left panel) and in a CD25 Isotype control (right panel). (G) P2X7 positive CD24^{+} CD127$^{-/low}$ cells gating (red arrow) from CD4^{++} CD25^{+} plot in a healthy control (left panel) and a septic patient (right panel).

Figure S6. Correlations between the percentage of CD3^{-}CD4^{+} cells and inflammatory cytokines in plasma and clinical markers. (A) Correlation between the percentage of CD3^{-}CD4^{+} cells and serum levels of IL-6 (left) and IL8 (right) cytokines, (B) or with clinical markers lactate (left) and Bilirubin (right) from septic patients at day 1. r Spearman test was used for non-parametrical distributed variables; each dot represents a single patient; *p< 0.05; **p< 0.01; ***p< 0.001.

Figure S7. Correlations between the percentage of CD3^{-}CD8^{+} cells and inflammatory cytokines in plasma or age of septic patients. (A) Correlation between the percentage of CD3^{-}CD8^{+} cells and serum levels of IL-6 (left) and IL8 (right) cytokines from septic patients at day 1, (B) or with the age of patients; r Spearman test was used for non-parametrical distributed variables; each dot represents a single patient; *p< 0.05; **p< 0.01.

Figure S8. Correlations between T cell populations. (A) Correlation between the percentage of P2X7 in CD4^{+}CD25^{++}CD127$^{-/low}$ cells and CD3^{+}CD4^{+} cells (B) or

$CD3^{+}CD8^{+}$ cells in healthy controls (left panel) and septic patients (right panel). r spearman test was used for correlations; each dot represents a single patient; *p< 0.05; *ns*, not significant difference (p> 0.05).

Figure S9. Correlations between the percentage of $CD4^{++}CD25^{+}CD127^{-/low}$ cells and bicarbonate of septic patients. r Spearman test was used for non-parametrical distributed variables; each dot represents a single patient; *ns*, not significant difference (p> 0.05).

Figure S10. Correlation between the percentage of PBMCs subsets and the percentage of their respective P2X7 expression. Correlation between the percentage of P2X7 expression and the cell proportion in (A) $CD3^{-}CD19^{+}$ cells, (B) $CD3CD19^{-}CD14^{-}CD16^{++}$ cells, (C) $CD3^{+}CD4^{+}$ cells, (D) $CD3^{+}CD8^{+}$ cells, and (E) $CD4^{++}CD25^{+}CD127^{-/low}$ cells. r Spearman test was used for non-parametrical distributed variables; each dot represents a single patient; *ns*, not significant difference (p> 0.05).

Figure S11. The increase of P2X7 receptor expression correlates in all PBMCs from septic patients but not in healthy controls. (A) Correlation between the percentage of P2X7 expression in $CD3^{+}CD8^{+}$, $CD3^{+}CD4^{+}$, $CD3^{-}CD19^{+}$, and $CD3^{-}CD19^{-}CD14CD16^{++}$ cells, and the P2X7 median intensity fluorescence (MFI) from $CD3^{-}CD14^{+}$ cells in septic patients; (B) and healthy controls. r spearman test was used for correlations; each dot represents a single patient; *p< 0.05; **p< 0.01; ***p< 0.001; *ns*, not significant difference (p> 0.05).

Figure S1

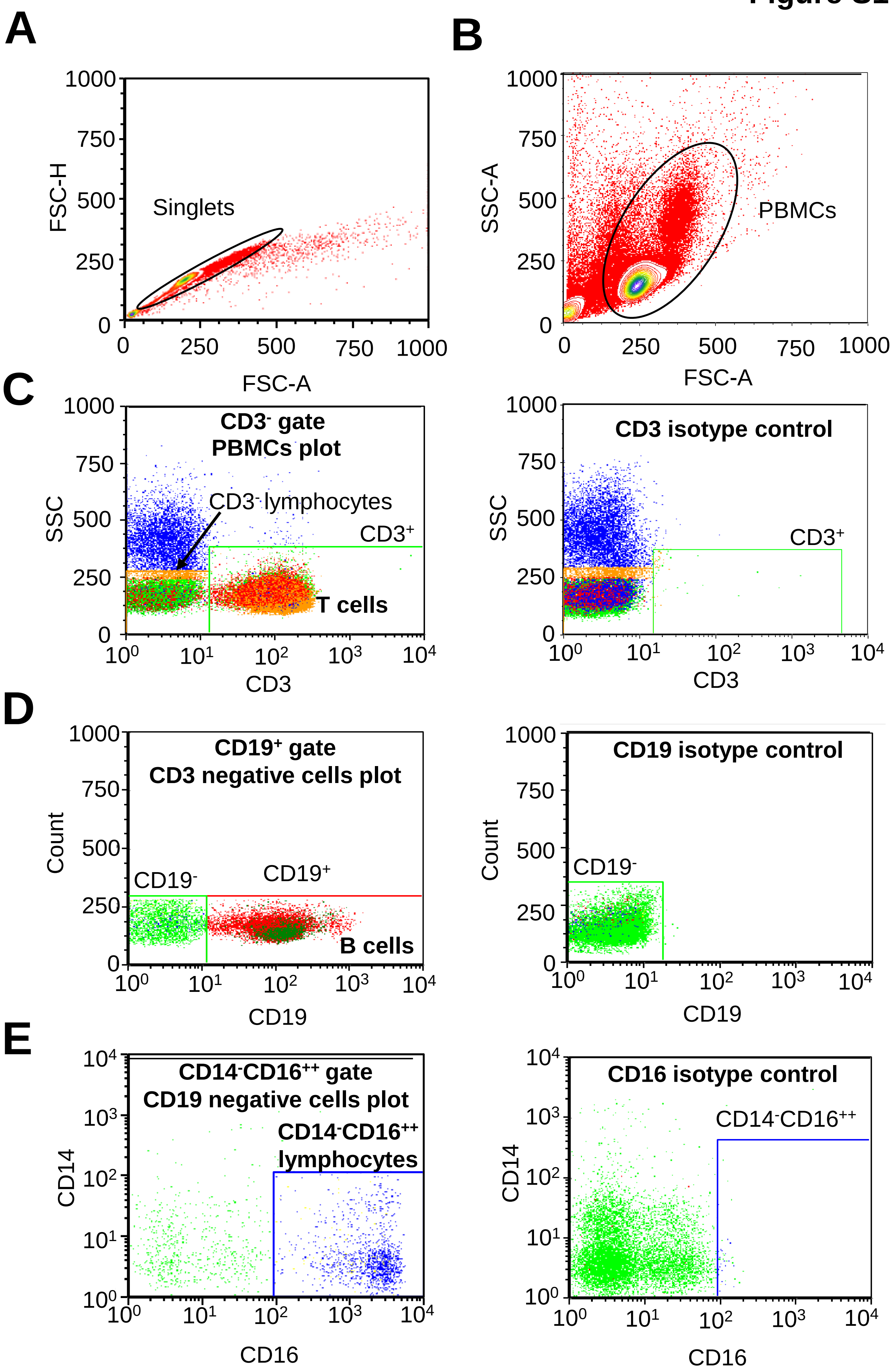
A
B
C
D
E
Singlets
FSC-H
FSC-A
PBMCs
SSC-A
CD3- gate
PBMCs plot
CD3- lymphocytes
CD3+
T cells
SSC
CD3
CD3 isotype control
CD19+ gate
CD3 negative cells plot
CD19-
CD19+
B cells
Count
CD19
CD19 isotype control
CD14-CD16++ gate
CD19 negative cells plot
CD14-CD16++
lymphocytes
CD14
CD16
CD16 isotype control

Figure S2

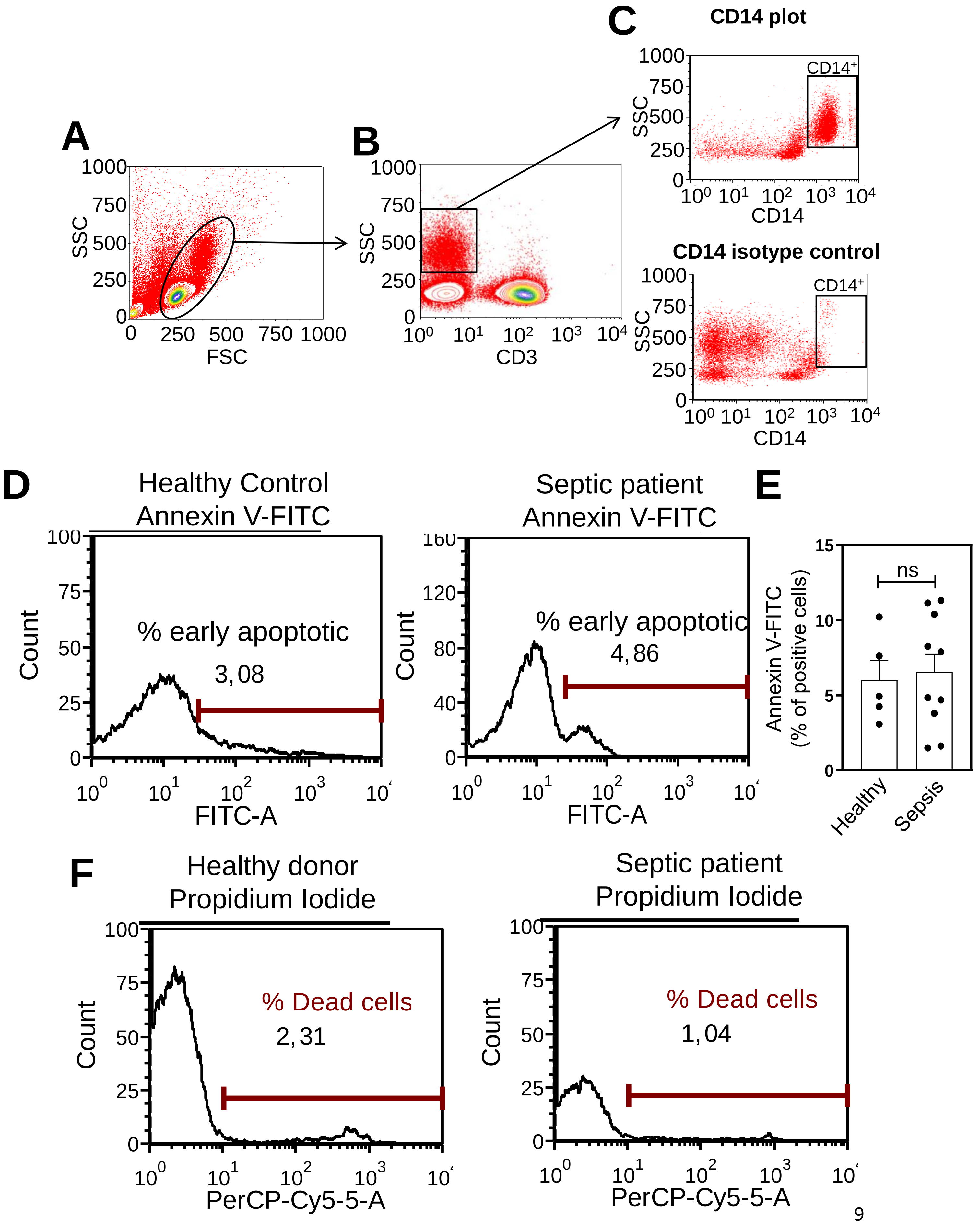
A
SSC
FSC
B
SSC
CD3
C
CD14 plot
CD14+
SSC
CD14
CD14 isotype control
CD14+
SSC
CD14
D
Healthy Control
Annexin V-FITC
Count
% early apoptotic
3,08
FITC-A
Septic patient
Annexin V-FITC
Count
% early apoptotic
4,86
FITC-A
E
Annexin V-FITC
(% of positive cells)
ns
Healthy
Sepsis
F
Healthy donor
Propidium Iodide
Count
% Dead cells
2,31
PerCP-Cy5-5-A
Septic patient
Propidium Iodide
Count
% Dead cells
1,04
PerCP-Cy5-5-A

Figure S3

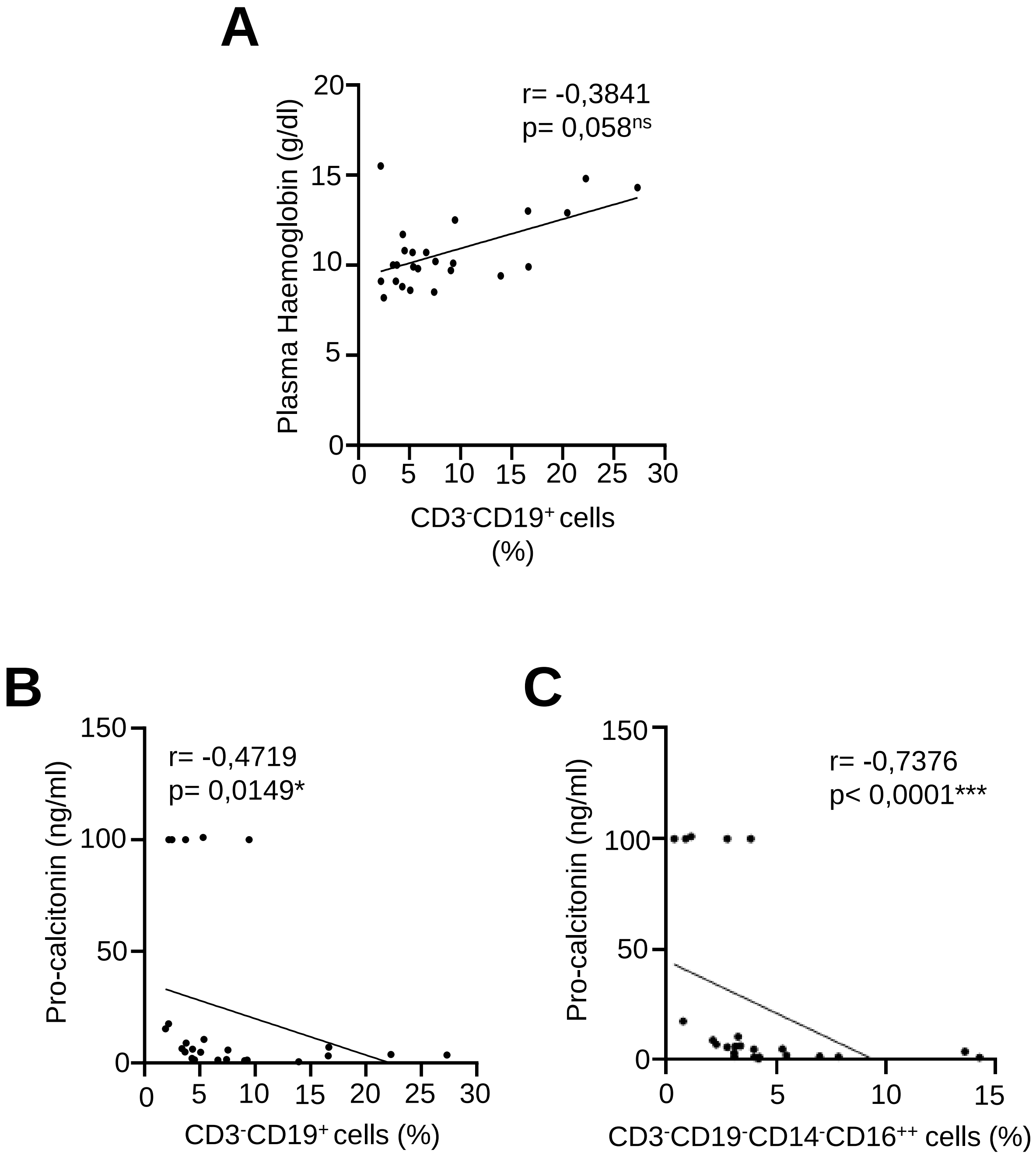
A
r= -0,3841
p= 0,058ns
Plasma Haemoglobin (g/dl)
20
15
10
5
0
0 5 10 15 20 25 30
CD3-CD19+ cells (%)
B
r= -0,4719
p= 0,0149*
Pro-calcitonin (ng/ml)
150
100
50
0
0 5 10 15 20 25 30
CD3-CD19+ cells (%)
C
r= -0,7376
p< 0,0001***
Pro-calcitonin (ng/ml)
150
100
50
0
0 5 10 15
CD3-CD19-CD14-CD16++ cells (%)

Figure S4

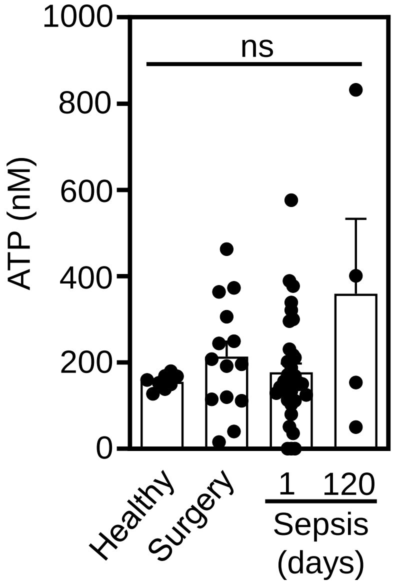
1000
800
600
400
200
0
ATP (nM)
ns
Healthy
Surgery
1
120
Sepsis
(days)

Figure S5

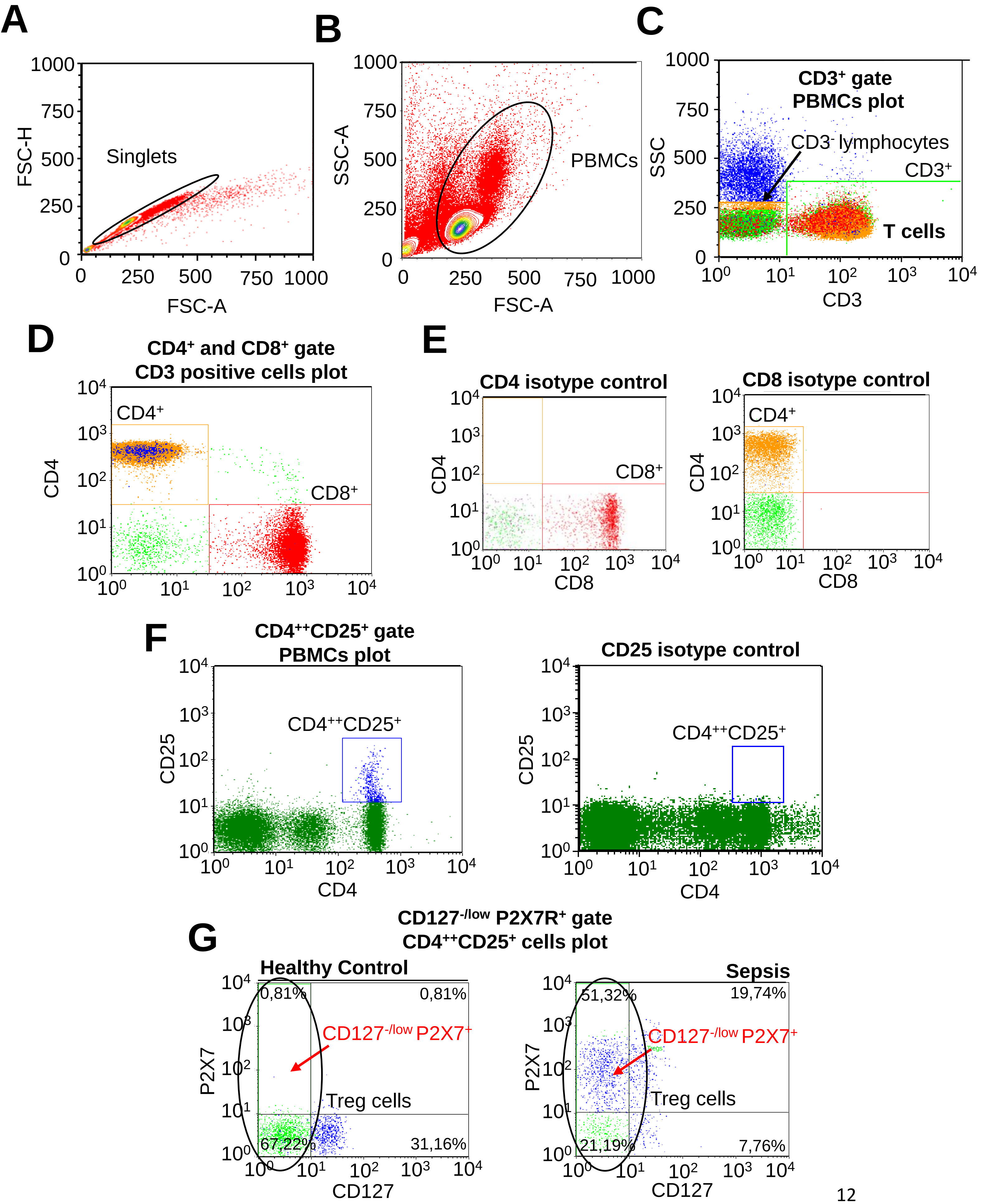
A
Singlets
FSC-H
FSC-A
B
PBMCs
SSC-A
FSC-A
C
CD3+ gate
PBMCs plot
CD3- lymphocytes
CD3+
T cells
SSC
CD3
D
CD4+ and CD8+ gate
CD3 positive cells plot
CD4+
CD8+
CD4
E
CD4 isotype control
CD8+
CD4
CD8
CD8 isotype control
CD4+
CD4
CD8
F
CD4++CD25+ gate
PBMCs plot
CD4++CD25+
CD25
CD4
CD25 isotype control
CD4++CD25+
CD25
CD4
G
CD127-/low P2X7R+ gate
CD4++CD25+ cells plot
Healthy Control
0,81%
0,81%
CD127-/low P2X7+
Treg cells
67,22%
31,16%
P2X7
CD127
Sepsis
51,32%
19,74%
CD127-/low P2X7+
Treg cells
21,19%
7,76%
P2X7
CD127

Figure S6

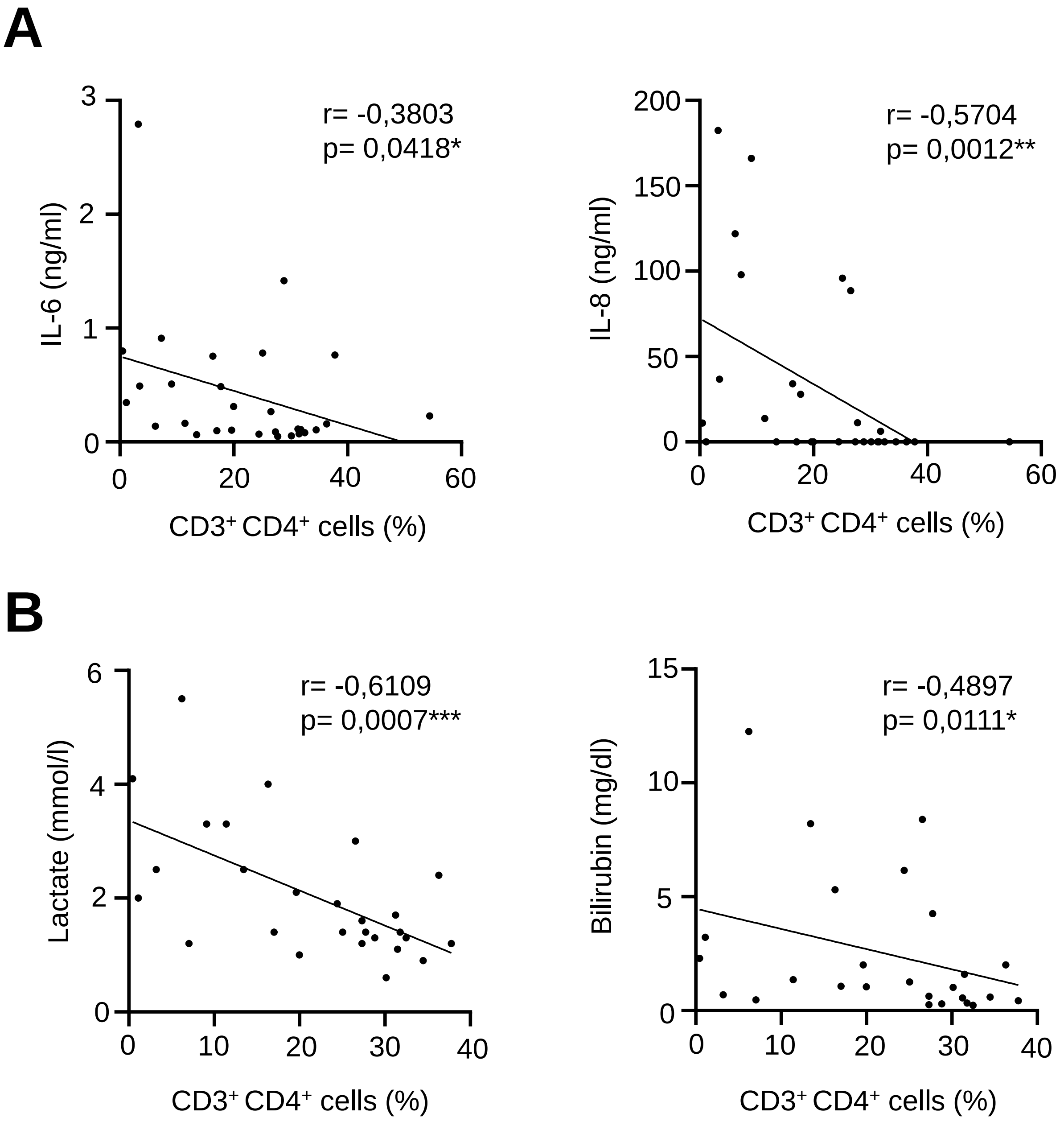
A
r= -0,3803
p= 0,0418*
IL-6 (ng/ml)
CD3⁺ CD4⁺ cells (%)
r= -0,5704
p= 0,0012**
IL-8 (ng/ml)
CD3⁺ CD4⁺ cells (%)
B
r= -0,6109
p= 0,0007***
Lactate (mmol/l)
CD3⁺ CD4⁺ cells (%)
r= -0,4897
p= 0,0111*
Bilirubin (mg/dl)
CD3⁺ CD4⁺ cells (%)

Figure S7

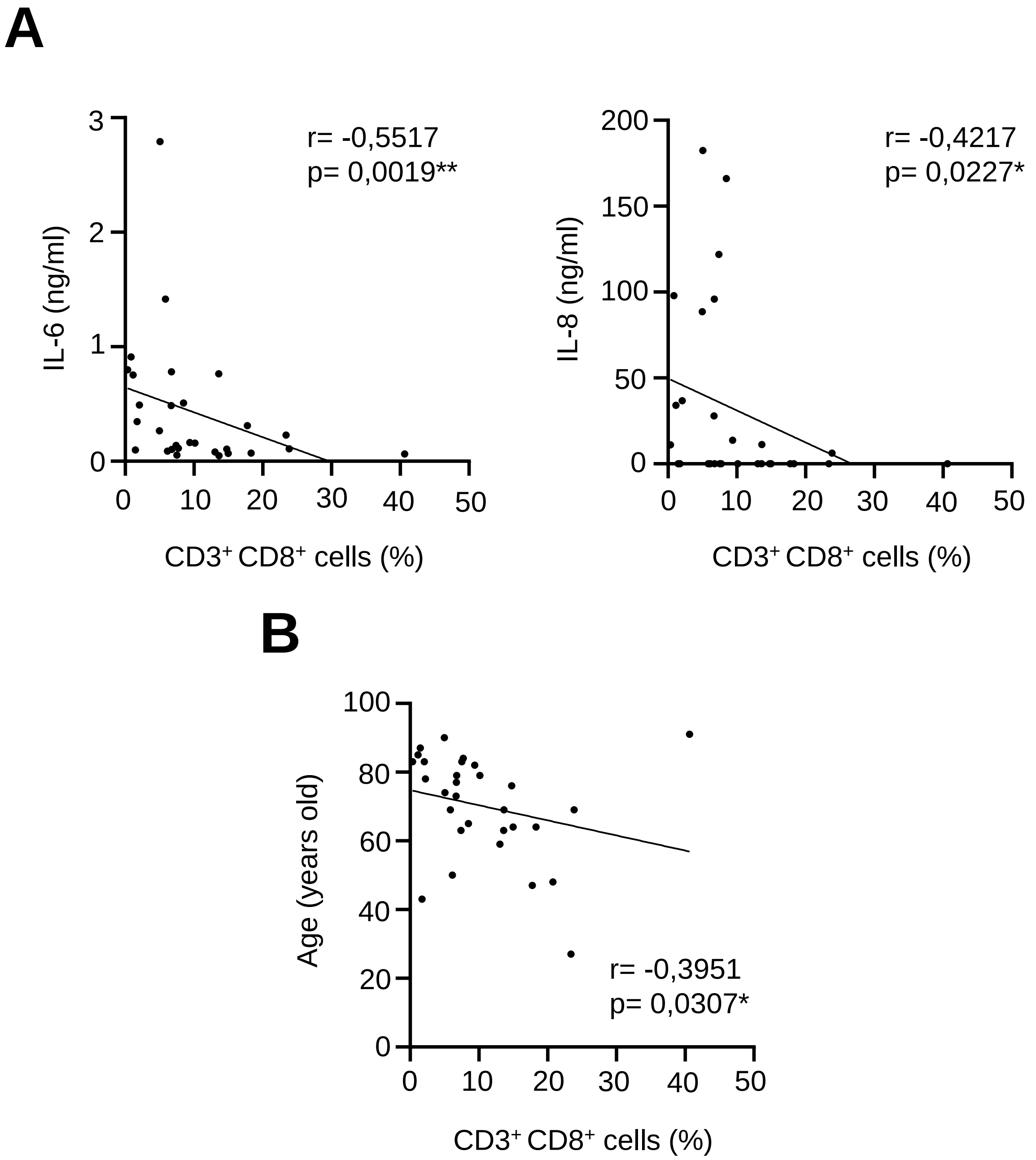
A
r= -0,5517
p= 0,0019**
IL-6 (ng/ml)
CD3+ CD8+ cells (%)
r= -0,4217
p= 0,0227*
IL-8 (ng/ml)
CD3+ CD8+ cells (%)
B
Age (years old)
r= -0,3951
p= 0,0307*
CD3+ CD8+ cells (%)

Figure S8

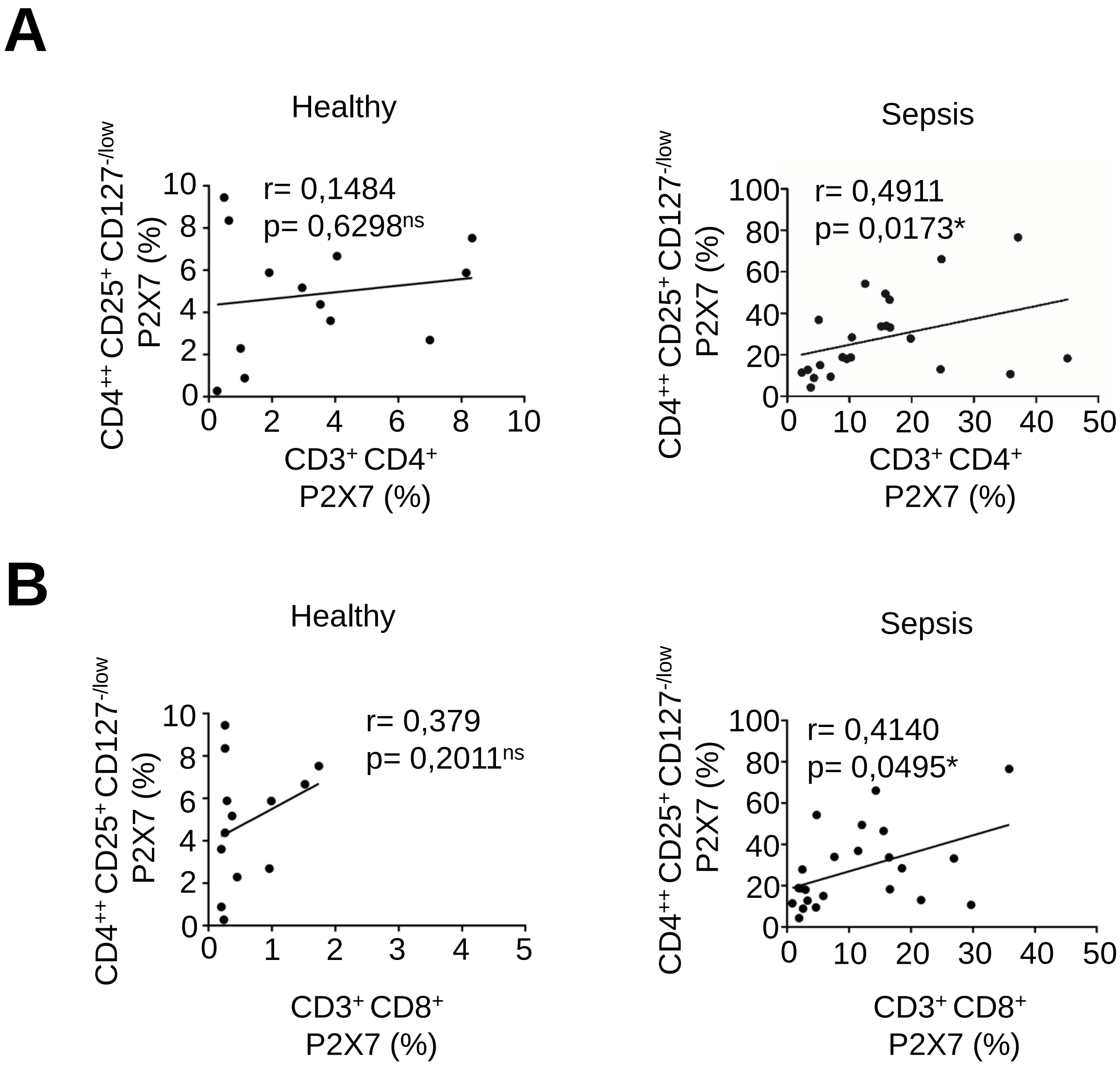
A
Healthy
r= 0,1484
p= 0,6298ns
CD4++ CD25+ CD127-/low P2X7 (%)
CD3+ CD4+ P2X7 (%)
Sepsis
r= 0,4911
p= 0,0173*
CD4++ CD25+ CD127-/low P2X7 (%)
CD3+ CD4+ P2X7 (%)
B
Healthy
r= 0,379
p= 0,2011ns
CD4++ CD25+ CD127-/low P2X7 (%)
CD3+ CD8+ P2X7 (%)
Sepsis
r= 0,4140
p= 0,0495*
CD4++ CD25+ CD127-/low P2X7 (%)
CD3+ CD8+ P2X7 (%)

Figure S9

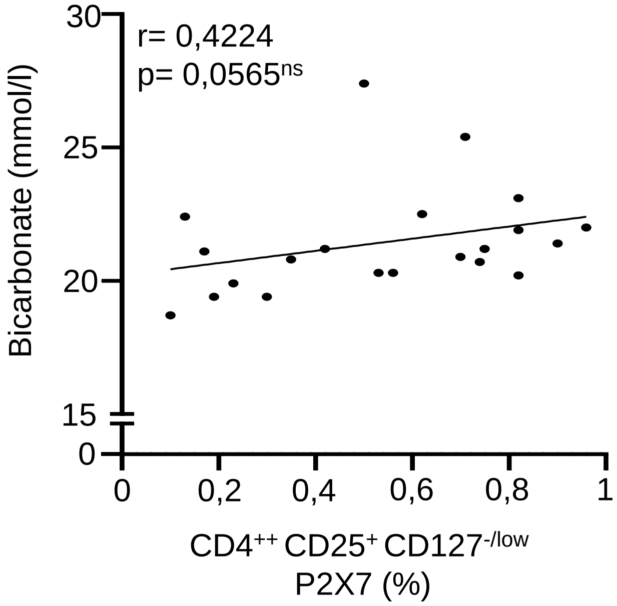
r= 0,4224
p= 0,0565ns
Bicarbonate (mmol/l)
30
25
20
15
0
0
0,2
0,4
0,6
0,8
1
CD4++ CD25+ CD127-/low
P2X7 (%)

Figure S10

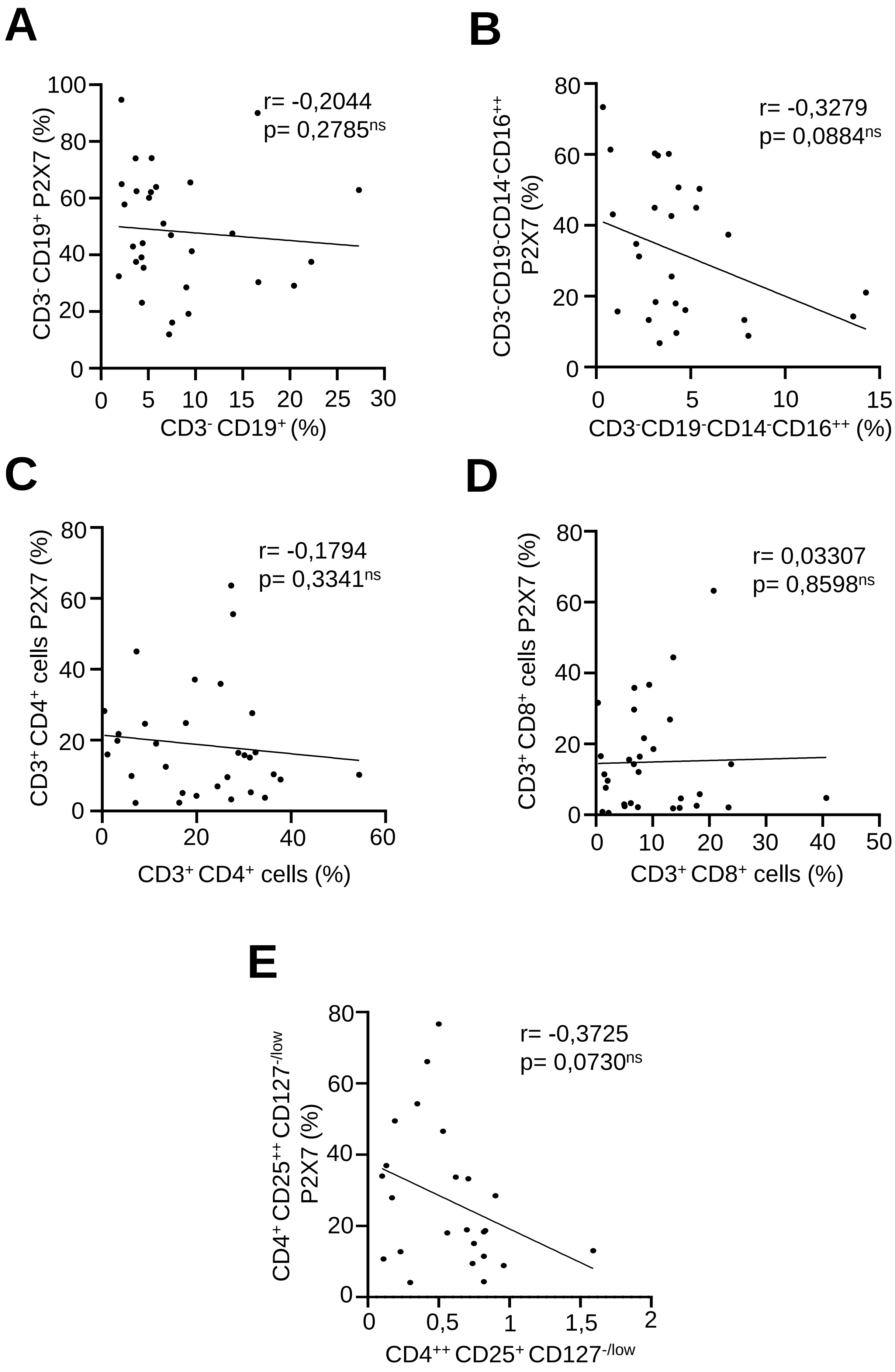
A
r= -0,2044
p= 0,2785ns
CD3- CD19+ P2X7 (%)
CD3- CD19+ (%)
B
r= -0,3279
p= 0,0884ns
CD3-CD19-CD14-CD16++ P2X7 (%)
CD3-CD19-CD14-CD16++ (%)
C
r= -0,1794
p= 0,3341ns
CD3+ CD4+ cells P2X7 (%)
CD3+ CD4+ cells (%)
D
r= 0,03307
p= 0,8598ns
CD3+ CD8+ cells P2X7 (%)
CD3+ CD8+ cells (%)
E
r= -0,3725
p= 0,0730ns
CD4+ CD25++ CD127-/low P2X7 (%)
CD4++ CD25+ CD127-/low cells (%)

Figure S11

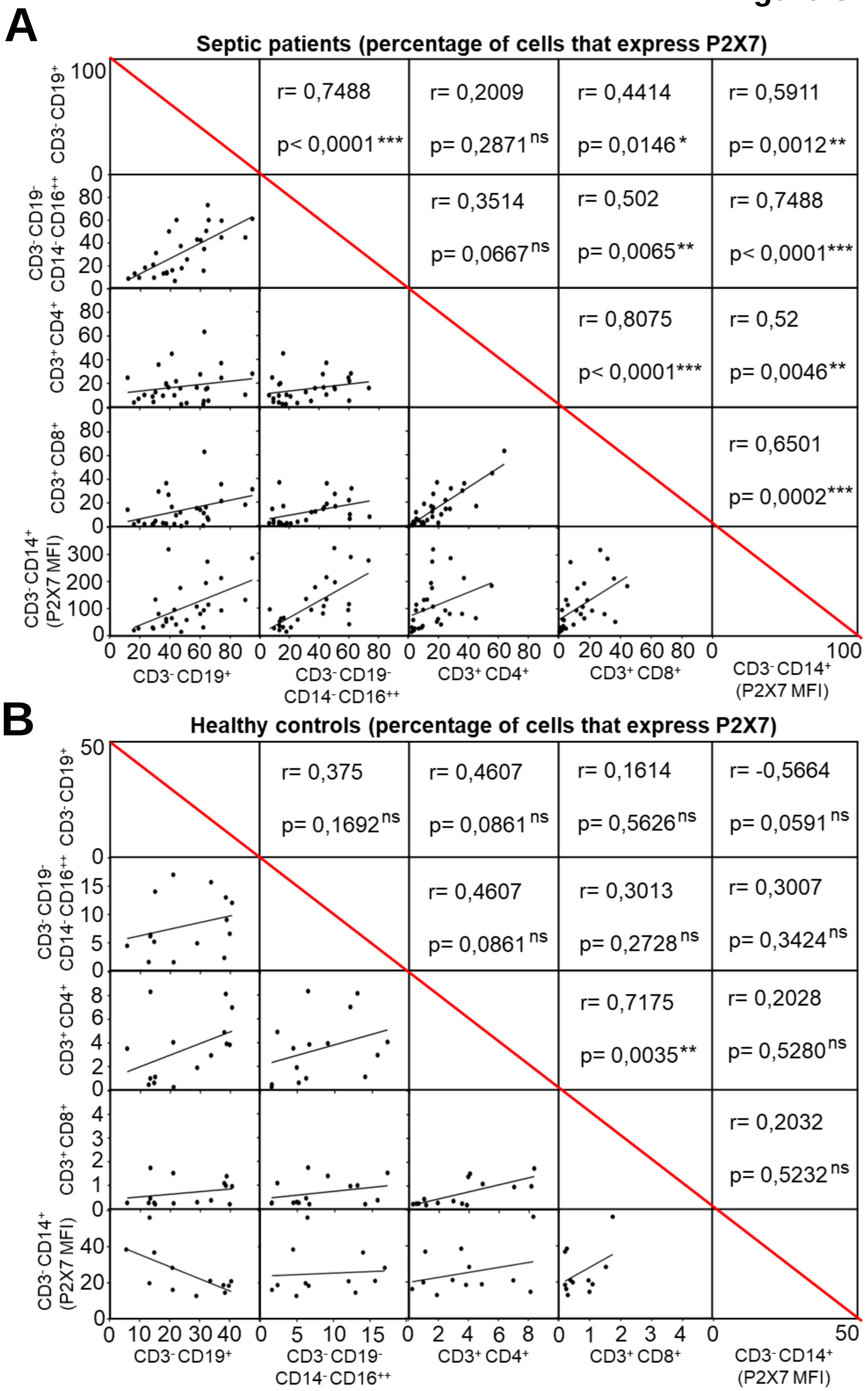
A
Septic patients (percentage of cells that express P2X7)
r= 0,7488
p< 0,0001***
r= 0,2009
p= 0,2871 ns
r= 0,4414
p= 0,0146*
r= 0,5911
p= 0,0012**
r= 0,3514
p= 0,0667 ns
r= 0,502
p= 0,0065**
r= 0,7488
p< 0,0001***
r= 0,8075
p< 0,0001***
r= 0,52
p= 0,0046**
r= 0,6501
p= 0,0002***
CD3- CD19+
CD3- CD19- CD14- CD16++
CD3+ CD4+
CD3+ CD8+
CD3- CD14+ (P2X7 MFI)
B
Healthy controls (percentage of cells that express P2X7)
r= 0,375
p= 0,1692 ns
r= 0,4607
p= 0,0861 ns
r= 0,1614
p= 0,5626 ns
r= -0,5664
p= 0,0591 ns
r= 0,4607
p= 0,0861 ns
r= 0,3013
p= 0,2728 ns
r= 0,3007
p= 0,3424 ns
r= 0,7175
p= 0,0035**
r= 0,2028
p= 0,5280 ns
r= 0,2032
p= 0,5232 ns
CD3- CD19+
CD3- CD19- CD14- CD16++
CD3+ CD4+
CD3+ CD8+
CD3- CD14+ (P2X7 MFI)
